# How to Prevent the Drop-Out: Understanding Why Adults Participate in Summative eHealth Evaluations

**DOI:** 10.1007/s41666-023-00131-8

**Published:** 2023-03-04

**Authors:** Marian Z. M. Hurmuz, Stephanie M. Jansen-Kosterink, Lex van Velsen

**Affiliations:** 1 Roessingh Research and Development, Roessinghsbleekweg 33B, 7522 AH Enschede, The Netherlands; 2 Biomedical Signal and Systems Group, University of Twente, Drienerlolaan 5, 7522 NB Enschede, The Netherlands

**Keywords:** eHealth, Participants, Research participation, Summative evaluation, Drop-out

## Abstract

The aim of this study was to investigate why adults participate in summative eHealth evaluations, and whether their reasons for participating affect their (non-)use of eHealth. A questionnaire was distributed among adults (aged ≥ 18 years) who participated in a summative eHealth evaluation. This questionnaire focused on participants’ reason to enroll, their expectations, and on whether the study met their expectations. Answers to open-ended questions were coded by two researchers independently. With the generalized estimating equations method we tested whether there is a difference between the type of reasons in use of the eHealth service. One hundred and thirty-one adults participated (64.9% female; mean age 62.5 years (SD = 10.5)). Their reasons for participating were mainly health-related (e.g., being more active). Between two types of motivations there was a difference in the use of the eHealth service: Participants with an intellectual motivation were more likely to drop out, compared to participants with an altruistic motivation. The most prevalent expectations when joining a summative eHealth evaluation were health-related (like expecting to improve one’s health). 38.6% of the participants said their expectation was fulfilled by the study. In conclusion, We encourage eHealth evaluators to learn about adults’ motivation to participate in their summative evaluation, as this motivation is very likely to affect their results. Including altruistically motivated participants biases the results by their tendency to continue participating in a study.

## Introduction

High drop-out among participants in eHealth studies, is a common problem (e.g. [1–3]) which impacts study results. For example, a study loses statistical power [4], and it becomes difficult to determine the effectiveness of eHealth services [5, 6]. When experiencing drop-out, it is important to investigate why this occurs. Maybe it can be prevented by adapting a study, adapting the eHealth service, or by giving better explanations to participants. When looking at summative eHealth evaluation (i.e. evaluation when an eHealth service is already developed and ready to be assessed for its effects and uptake [7, 8]) reports, they do not disclose reasons for drop-out rates (e.g. [9–13]), or provide short explanations, like a loss of contact [14], participants moving house [15, 16], personal/family reasons [14, 17, 18], studies being too time consuming [19], participants being too busy or with a lack of time [14, 16], being reluctant towards using technology [19], technical problems [18], not wanting to be confronted with a medical condition [19], or medical problems [14, 16, 18]. However, these are all merely short explanations and reasons for dropping-out are often not being reported, potentially not even investigated in-depth. A first step in reducing the number of drop-outs in eHealth use in summative eHealth evaluations is to examine participants’ reasons for participating.

A lot of research focusing on motivations of different groups to participate in health studies has been conducted. Soule and colleagues [20] studied, among 164 patients suffering from heart diseases, the importance of four different motivations (intellectual motivation, altruistic motivation, health motivation, and financial motivation) to participate in observational health research. They found that the most important reason to participate was altruistic: Participants wanted to help future patients in the same situation, or to help the researchers. The least important motivation, they found, was financial. In another study conducted in Canada, 39 adults were interviewed about reasons for participating in different kinds of health studies. These adults, it turned out, primarily participated for their own health gain: to have access to drugs, to have access to healthcare, and to have access to technologies for monitoring their health. Also in this study, receiving a financial incentive was not a pre-dominant motivation [21]. Furthermore, Bouida and colleagues [22] investigated among patients, healthy volunteers and doctors in Tunisia reasons for enrolling in clinical trials. This population mentioned two main reasons. The first one was related to altruism, and the second reason was that they thought it is important to contribute to improving the healthcare. All three studies suggest that, in healthcare, adults primarily participate in studies to either help themselves or others.

For the context of eHealth, studies that uncover reasons for participating in summative evaluations among adults are scarce. Coley and colleagues [23] studied reasons for participating in a randomized controlled trial (RCT) involving an eHealth service focusing on prevention of cardiovascular diseases among older adults in Finland, France and the Netherlands. The top three main motivations for participants to take part were contributing to science, improving one’s lifestyle to improve health, and obtaining additional medical monitoring. However, a limitation of this study is that it was about participating in an RCT, which is a highly controlled study [24]. This could have influenced older adults’ willingness to participate. In another study we found, James and colleagues [25] investigated facilitators to participate in eHealth studies among African American women. They found that being interested in the topic, wanting to be more educated about the topic and contributing to the greater good were the three most mentioned facilitators. However, they asked women to complete a survey and think about the facilitators for participating in eHealth studies in general. These women did not actual participate in an eHealth evaluation. Finally, we also found an article focusing on employees’ reasons to participate in an eHealth intervention in their workplace in the United Kingdom (UK) [26]. So, this article is looking at an eHealth intervention which is implemented in different UK worksites, and not at an eHealth evaluation. However, we still looked into those reasons which were mostly related to improving their own health or to liking the intervention. Next to these, recommendations of other colleagues was also a reason to participate. Taking the prior literature into consideration, we still need more studies with a diverse range of eHealth services. Because now it does not focus on summative eHealth evaluations, even though in these type of evaluations researchers struggle with high drop-out rates. By gaining knowledge on participants’ motivation to participate in a summative eHealth evaluation, we can better explain the high drop-out rates in eHealth use in summative evaluations and we can tune their setup towards the participants’ needs and try to reduce the number of drop-outs. So, in this article, we report on a study in which we investigated adults’ motivations to participate in different summative eHealth evaluations, conducted in real-world settings, and tested whether their reasons affect the (non-)use of eHealth.

## Methods

### Study Design

To answer the following research question “What are adults’ motivations to participate in summative eHealth evaluations, and does these reasons affect the (non-)use of eHealth?”, we conducted a cross-sectional study. By having participants completing one questionnaire at a specific moment, we were able to answer our research question. The specific moment they completed the questionnaire was after they participated in a summative eHealth evaluation. Within three studies in which different eHealth services were evaluated, participants were asked to complete this online questionnaire about their reasons to participate and their expectations of the study. With this information we could answer the research question under investigation. The participants completed the questionnaire directly after they finished the study or directly after they dropped out. All three studies were conducted in the Netherlands.

### eHealth Services

**Fig. 1:**
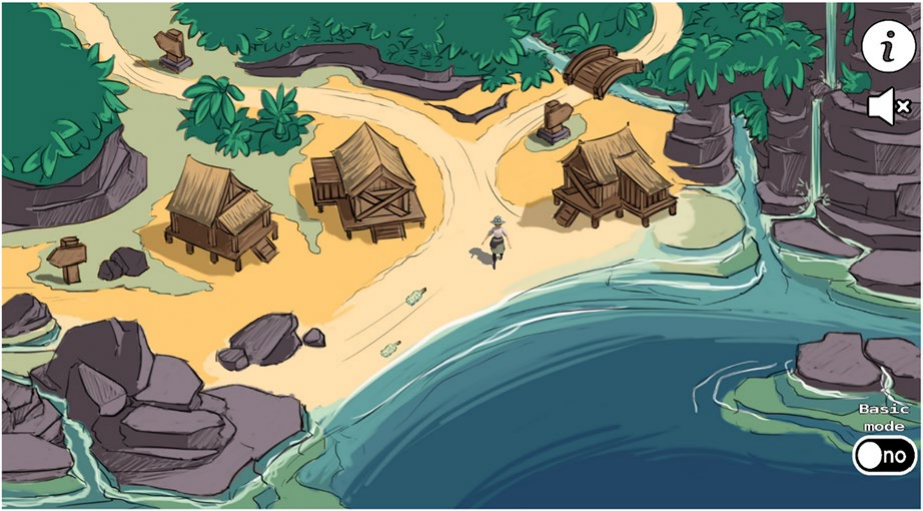
Screenshot of eHealth service Stranded

**Fig. 2:**
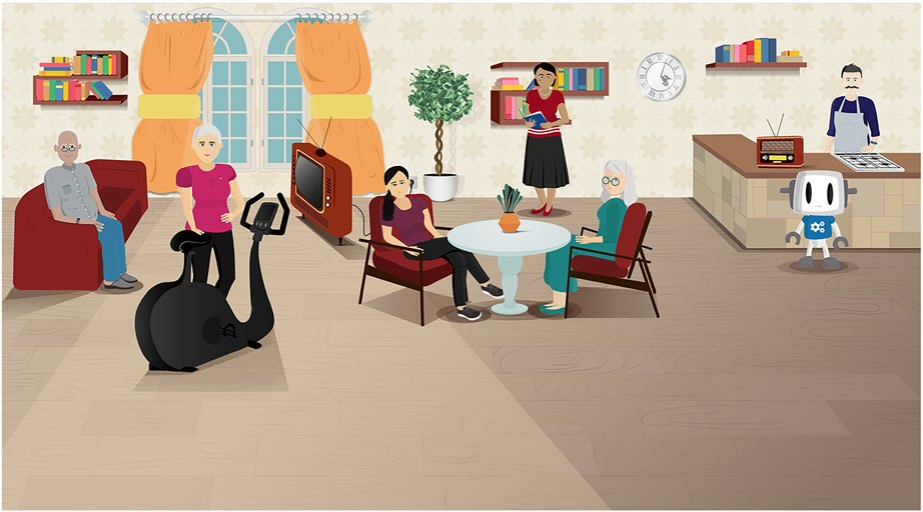
Screenshot of eHealth service Council of Coaches. (Names of the virtual coaches f.l.t.r.: Carlos (peer), Olivia (physical activity coach), Emma (social coach), Katarzyna (diabetes coach), Helen (cognitive coach), Coda (helpdesk robot), François (nutrition coach))

**Fig. 3:**
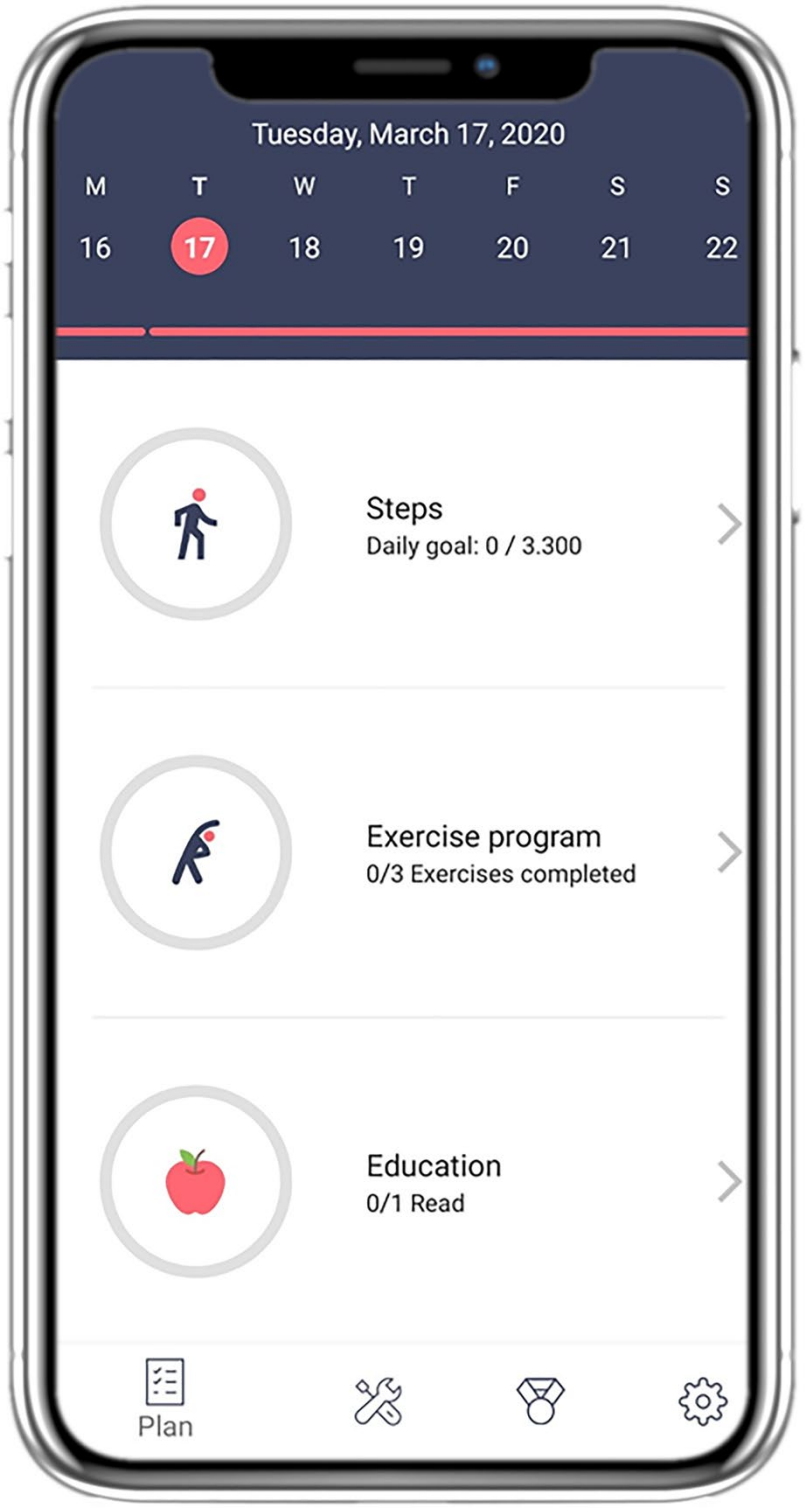
Screenshot of eHealth service selfBACK app (showing weekly self-management plan)

Motivations to participate in the evaluation of three different eHealth services were inventoried. The first service, Stranded (see Fig. 1), is a web-based, gamified eHealth service for (pre-)frail older adults. Stranded [27] consists of two parts: a falls prevention programme based on the OTAGO Programme [28], and cognitive minigames. The falls prevention programme consists of physical exercise videos that older adults can perform at home. These exercises focus on improving muscle strength, balance, and flexibility. The minigames are different kinds of puzzle games. The duration of the study evaluating Stranded was four weeks. The second eHealth service, Council of Coaches (COUCH) [29] (see Fig. 2), is a web-based service designed for adults with Diabetes Mellitus Type 2 or Chronic Pain, and older adults who are dealing with age-related impairments. The goal of COUCH is to encourage a healthy lifestyle via conversations with virtual coaches. Within COUCH six different coaches are available: a physical activity coach, a nutrition coach, a social coach, a cognitive coach, a chronic pain coach (only available for users with chronic pain), and a diabetes coach (only available for users with diabetes). During the summative evaluation of COUCH, participants could use the eHealth service for four weeks. The last eHealth service, the selfBACK app [30–32] (see Fig. 3), is a mobile self-management application for adults with neck and/or low back pain. The selfBACK app provides users with a weekly tailored plan to self-manage this pain. The weekly plain focusses on three aspects: Physical activity (i.e., daily step data), physical exercises to strengthen the muscles and increase flexibility, and educational messages to motivate users and to give them advice. This study with the selfBACK app lasted for six weeks.

### Study Population

The study population of our study were the participants of different eHealth evaluations. Within the Stranded and COUCH evaluations, the participants were 55 years of age or older and able to speak and read Dutch. Within the selfBACK app evaluation, the participants were 18 years or older with neck and/or low back pain and able to speak and read Dutch. Participants were recruited via mass mailing and advertisements in newspapers and on social media.

### Data Collection

An online questionnaire was distributed, consisting of seven questions (see Appendix A for full questionnaire). First, two questions on demographics (age and gender), and one multiple choice question, inventorying how participants came across the study (e.g., advertisement in local newspaper, social media, friend/family/colleague). Then, there was one open question, asking why participants wanted to participate in the study. Finally, to have more in-depth information about expectations towards summative eHealth studies that participants have, we posed three more questions. These questions elicited participants’ initial expectations of the study (open question), asked whether the study met these expectations (closed question yes/no), and questioned why the study did (not) meet their expectations (open question).

### Data Analyses

We calculated descriptive statistics (frequency, mean, standard deviation, percentages) within SPSS v.19 to describe the demographics, to describe how participants came across the summative evaluation, and to inventory whether the evaluation met their expectations. Two authors (MH and SJK) coded all open-ended questions thematically. Here, we used a deductive approach to code the reasons for participating in a study. The themes by Soule and colleagues [20] were used as the initial codebook: Intellectual motivation (i.e., being interested in the study), altruistic motivation (i.e., helping researchers and/or future patients), health motivation (i.e., wanting to improve one’s health), financial motivation (i.e., receiving compensation (which does not need to be necessarily a monetary compensation)), and other motivations (e.g., fun, gaining knowledge). We used an inductive approach to code the other two open-ended questions (what were the expectations, why the study did (not) meet these expectations). The first and second authors (MH and SJK) coded all answers separately, and then discussed them together until there were no disagreements left.

To test for differences between the different motivation types, we conducted logistic regression analyses according to the generalized estimating equations (GEE) method [33] within SPSS. The dependent variable was whether or not the participants used the eHealth service during the length of the study; predictors were the types of motivations. We opted for the GEE method, as some participants mentioned multiple reasons for participating. To be able to compare all three motivations (altruistic motivation versus intellectual motivation, altruistic motivation versus health motivation, and intellectual motivation versus health motivation), we performed the GEE analysis twice with different reference categories. After these analyses, we corrected the p-values according to the Holm-Bonferroni method [34]. We excluded the category ‘other motivation’ from these analyses, as this was a relatively small, heterogeneous group of reasons that did not make for a sensible collection.

### Ethics

All studies were conducted according to the principles of the Declaration of Helsinki (64th WMA General Assembly, Fortaleza, Brazil, October 2013) and in accordance with the Medical Research Involving Human Subjects Act (Dutch law). The Medical Research Ethics Committee CMO Oost-Nederland stated that these studies do not require formal medical ethical approval (file numbers: 2019–5296, 2019–5555, 2020–6501). All participants signed an informed consent form before participating.

## Results

A total of 131 adults completed the questionnaire. Their mean age was 62.5 years (SD = 10.5); 64.9% was female. Fifty-three participants took part in the Stranded evaluation, 49 evaluated COUCH, and 29 evaluated the selfBACK app. Most participants came across the studies via advertisements in local newspapers (66.4%). From 101 adults of the total study population, we have data whether they continued using the eHealth service during the full length of the study. Of these participants, just over half of the study population used the eHealth service during the full length of the study: 55 out of 101 adults (54.5%). Table 1 shows the distribution of the demographics, data regarding how participants were recruited and data regarding use of the eHealth service of the different groups.

**Table 1:** Demographics and descriptive statistics for completed study of the total study population, and divided into the three eHealth groups

		Total group (*N* = 131)	Stranded group (*N* = 53)	COUCH group (*N* = 49)	selfBACK app group (*N* = 29)
Age (Range M (SD))		23 – 87 62.5 (10.5)	55 – 84 64.4 (6.3)	55 – 87 65.4 (7.5)	23 – 77 53.9 (15.7)
Gender (*N* | %)	*Male*	46 | 35.1%	17 | 32.1%	14 | 28.6%	15 | 51.7%
	*Female*	85 | 64.9%	36| 67.9%	35 | 71.4%	14 | 48.3%
Recruited via … (*N* | %)	*Advertisement newspaper*	87 | 66.4%	28 | 52.8%	35 | 71.4%	24 | 82.8%
	*Advertisement social media*	9| 6.9%	4 | 7.5%	4 | 8.2%	1 | 3.4%
	*Friend/family/ colleague*	25 | 19.1%	11| 20.8%	10 | 20.4%	4 | 13.8%
	*Email research panel*	8 | 6.1%	8| 15.1%	-	-
	*Other*	2 | 1.5%	2| 3.8%	-	-
Continued using eHealth service for length of study (*N* | %)	*Yes*	55 | 42.0%	20 | 37.7%	11 | 22.4%	24 | 82.8%
	*No*	46 | 35.1%	6 | 11.3%	38 | 77.6%	2 | 6.9%
	*Missing*	30 | 22.9%	27 | 50.9%	-	3 | 10.3%

### Reasons to Participate

In total, 129 participants gave one or more reason(s) for participating in an evaluation, with a total of 157 reasons. Most of these reasons were related to health motivation (N = 81). Examples of these reasons are that they want to improve/maintain their health, to live a healthy life, to have more energy, to relieve their pain, or to be more physically active.

“*Because of an often found disease in the family, Type 2 Diabetes, I find it important to take my responsibility regarding my lifestyle.*” (P-100, female, 62 years, COUCH study).

“*The older you get, the more attention you need to pay to your physical health. This requires discipline and at the same time the ability to keep it together. I saw the exercises you provided as an opportunity to strengthen this.*” (P-32, male, 76 years, Stranded study).

The second most mentioned motivation was intellectual motivation (*N* = 41), followed by altruistic motivation (*N* = 22), and other motivations (*N* = 13). No participant gave a financial motivation to participate in these studies. Reasons related to intellectual motivation were, for example, being interested in the study or being curious about the eHealth service under investigation.

“*Out of curiosity. I wanted to know what kind of exercises such a programme offers. Whether it is useful for me. Whether it is fun. Why exercises and games are implemented in one programme?*” (P-39, female, 79 years, Stranded study).

Regarding their altruistic motivation, participants said they wanted to help the research(er) or wanted to help improve healthcare for future older adults/patients.

“*Because I think that if you want to develop new tools, technologies or drugs, you also need people who are willing to act as ‘guinea pigs’.*” (P-27, female, 59 years, Stranded study).

Other motivations participants mentioned for participating in these studies were: just for fun (*N* = 5), wanting to be introduced to eHealth (*N* = 5), because peers motivated them to participate (*N* = 2), and because of the reputation of the research centre (*N* = 1).

Table 2 shows the number of participants who used the eHealth service during the full length of the study and those that abandoned using the service, per motivation type. The statistical analyses show a clear difference in the degree of eHealth service use between participants with an altruistic motivation and participants with an intellectual motivation (see Table 3). The risk that participants drop out is 12.2 times higher among those with an intellectual motivation compared to those with an altruistic motivation (*P* = 0.042, 95%-CI = 1.648 – 90.827).

**Table 2:** Cross table showing number of times (not) continued use of eHealth service per motivation type

Type of motivation	Number of participants who used eHealth service during length of study	Number of participants who abandoned use of the eHealth service	Totals
Intellectual motivation	*N* = 17	*N* = 16	*N* = 33
Altruistic motivation	*N* = 13	*N* = 1	*N* = 14
Health motivation	*N* = 37	*N* = 29	*N* = 66
Totals	*N* = 67	*N* = 46	*N* = 113^a^

^a^ *N* = 113, because some participants had two different reasons to participate in the study

**Table 3:** Results logistic regression according to GEE method

Comparison	Odds ratio	95% Confidence Interval	*P*-value	Corrected *P*-value
Altruistic^a^ x Intellectual	12.2	1.65 – 90.8	0.014	0.042
Altruistic^a^ x Health	10.2	1.28 – 80.9	0.028	0.056
Intellectual^a^ x Health	0.83	0.40 – 1.73	0.624	0.624

^a^ Motivation category used as reference value

The underlined entries are the significance values

### Expectations for the eHealth Evaluation

When asking the participants about their initial expectations for the eHealth evaluation, 70 participants mentioned at least one expectation (with a total of 79 expectations), 39 participants indicated they had no expectations, 16 participants did not answer this question properly (i.e., not providing an expectation, but mentioning something else), and the remaining 6 participants only indicated that their expectations were (too) high. Most expectations were health-related (*N* = 41), followed by content-related (*N* = 34), and technology-related expectations (*N* = 4).

The health-related expectations can be divided into four kinds: Expecting to improve one’s health (*N* = 28), expecting to perform physical exercises (*N* = 6), expecting to become aware of one’s lifestyle (N = 5), and expecting to maintain one’s health (*N* = 2).

*“I expected to receive some exercises that might relieve my neck pain in some cases.”* (P-110, female, 33 years, selfBACK study).

Content-related expectations were divided into six kinds: Expecting to receive help/tips (*N* = 15), expecting to receive a positive prompt or nudge (*N* = 7), expecting to receive personalised content (N = 6), expecting to receive a combination of exercises and games (*N* = 3), expecting to receive a lot of content (*N* = 2), and expecting to be talking to real coaches (*N* = 1).

“*My expectation was that I would receive a personalised exercise programme […].*” (P-109, male, 34 years, selfBACK study).

Finally, technology-related expectations were either that participants thought the eHealth service was easy to use (*N* = 2), or that the eHealth service had a high maturity level (*N* = 2).

“*Beforehand, I thought it would be a simple programme, easy to start and fun to use as a variation.*” (P-48, female, 62 years, Stranded study).

Of the 70 participants who mentioned a specific expectation, 27 indicated that participating within the study fulfilled their expectation(s) (38.6%). Twenty-two participants gave a reason why their expectation(s) was/were fulfilled. This was either content-related (*N* = 13) (e.g., the eHealth service had suitable content, users received a positive prompt/nudge from the eHealth service), health-related (*N* = 8) (e.g., improved health state), or personal (*N* = 1) (enjoyed the eHealth service). The 43 participants whose expectations were not fulfilled, all explained their answer. The most mentioned reason was content-related (*N* = 29) (e.g., lack of specific or personalised content), followed by personal reasons (*N* = 9) (e.g., no fit with technology, lack of time), health-related (*N* = 7) (no improvement in health state), or technology-related (*N* = 7) (e.g., experienced problems while using the technology).

## Discussion

In this paper, we investigated the reasons of adults to participate in summative eHealth evaluations in real-world settings, and tested whether their reasons affect the degree to which they used the eHealth service during the study. Finally, we elicited participants’ expectations when joining these evaluations and assessed whether these expectations were met.

With regard to reasons for participating in summative eHealth evaluations, our findings show that most adults participate in order to actively do something for their own health state (e.g., improving their fitness levels, relieving pain). Townsend and Cox [21] also found that health-related reasons to participate in health studies are dominant. However, based on other prior literature (e.g., [20, 22, 23]), we expected that altruism would be (one of) the most prevalent reason to participate in summative eHealth evaluations. In our study, this reason was only a minor driver for participation. Furthermore, in our study, financial motivation was not mentioned by any participant as a reason to participate in a summative eHealth evaluation. It should be noted though, that in none of the studies there was a substantial financial compensation; the participants knew they would receive a small gift to thank them for their participation. Apparently this did not influence their reason to participate in the study. The literature shows a different picture. Here, financial incentives *are* one of the reasons to participate [25, 35, 36]. Explanations for the differences in the reasons for participating that we identified and those found in other studies, could be attributed to the use of the term ‘small gift’ in our information letters, or the different healthcare systems in the countries in which the studies were performed. After all, whether or not to participate in a health study when being in a healthcare system where every citizen is fully insured for a low fee (like in the Netherlands) might lead to a different incentive than when one lives in a country where being insured is less self-evident (like in the United States). In all, these results imply that during the recruitment process, potential participants should be primarily informed about the role the evaluation or the intervention can play with regard to their own health.

When analysing whether the reason to participate affected use of the eHealth service, we saw a difference in use between altruistically and intellectually motivated participants. Intellectually motivated adults are more likely to discontinue use of an eHealth service before the end of a study compared to altruistically motivated participants. In a time where optimizing adherence is a hot topic (some people even talk about an ‘engagement crisis’), we think this is an important finding. In order to further our understanding of adherence, studying the role of motivation is not new. Other researchers have, for example, studied the role of personal motivation types for complying with persuasive eHealth functionality [37]. We propose that in future evaluations focusing on eHealth use, researchers identify participants’ motivations at the beginning of the study. Later, they can then use this motivational profile to explain drop-outs and eHealth service use. The usefulness of this data would be enhanced by knowing the motivational profile of the addressable market for an eHealth service, so that the generalizability of the evaluation results can be made insightful.

Finally, our findings show that the expectations adults have about summative eHealth evaluations are mostly health-related or content-related. They expect that by participating in these studies, they will improve their health state, and receive helpful, personalised advice. Other studies also found that participants expect to receive this type of personalised content and these health benefits (e.g. [38–40]). When developing eHealth services with involvement of end-users, end-users often mention personalised content as an important factor (e.g. [41, 42]). In order to increase the success of a recruitment strategy, evaluators should therefore stress the health potential of taking part in the study and the eHealth service, and, if applicable, should stress the personalised features of the technology.

### Study Limitations

Our study has some limitations. First of all, in the three included studies, participants were recruited via self-enrolment. As a result, participants may have been motivated to participate in eHealth evaluations more than if we could have picked participants from the population at random. Possible, this has biased our results somewhat. Second, we chose to ask the study population after participation why they chose to participate and which expectations they had before starting the study. There is a possibility that participants were not sure about their initial reasons anymore, or their answers might have been affected by the study and by the eHealth service used. However, we do not think this had a major impact on the results, because of the comprehensive answers participants gave, and because there was no participant that mentioned (s)he was unable to recall his or her reasons. To confirm our findings, we propose that future summative eHealth evaluations identify participants’ reasons and expectations before starting. Third, during the primary eHealth evaluation there were some participants lost to follow-up. These participants did also not complete the questionnaire used in the current paper. We need to take in mind that this could have an affected the findings and their generalizability. Finally, our study was conducted in the Netherlands. We think that the healthcare system of the country participants live, influences the findings. In the Netherlands, residents have relatively good access to healthcare, as everyone has an healthcare insurance, and the general practitioner acts as a gatekeeper [43]. As it is easy to access healthcare for free in the Netherlands, we think that reasons such as ‘participating in study to gain access to healthcare’ do not play a role among our participants, or only marginally. So, the conclusions we can draw with our findings, do not directly apply to other countries with other healthcare systems.

## Conclusions

Drop-outs are a concern in science, in medical studies, and in summative eHealth evaluations. It is in the researchers’ interests to minimize the number of drop-outs in a study and to understand the reasons of the persons who decide to stop in an evaluation. For the case of summative eHealth evaluations, recruitment strategies should be focused on stressing the potential health benefits of participating in an evaluation and using the eHealth service. Offering a small monetary compensation will probably not benefit recruitment in a study where it is based on self-enrolment. Additionally, if the eHealth intervention offers personalised information or advice, this should be stressed in recruitment strategies, as participants appreciate such a feature. Using this strategy could probably result in a higher number of participants among those who expect personalisation, as their expectation will be confirmed. Altogether, researchers need to keep in mind when recruiting study participants, that those participants still represent the target population of the eHealth service under study. The strategies given here should not alter the representativity of the population.

## Data Availability

The data that support the findings of this study are available from the corresponding author, MH, upon reasonable request.

## Appendix A

**Questionnaire [English version translated for aim of this paper by first author (MH)]**

1. What is your gender? [male / female]
2. What is your age?
3. Through which channel did you find out about this study? [advertisement in local newspaper / advertisement on social media / flyer / friend family colleague / email from research panel / other channel]
4. Why did you want to participate in this study?
5. What were your expectations prior to this study?
6. Have these expectations been met? [yes / no]
7. Please indicate why this study has fulfilled your expectations / Please indicate why this study has not fulfilled your expectations

**Questionnaire [Dutch version which was distributed among the participants]**

1. Wat is uw geslacht? [man / vrouw]
2. What is your age?
3. Via welk kanaal bent u in aanraking gekomen met dit onderzoek? [krant / social media / flyer / vriend familie collega / e-mail van onderzoekspanel / anders]
4. Waarom wilde u deelnemen aan dit onderzoek?
5. Wat waren uw verwachtingen vooraf aan het onderzoek?
6. Is er voldaan aan deze verwachtingen? [ja / nee]
7. Geef aan waarom het onderzoek aan uw verwachtingen heeft voldaan / Geef aan waarom het onderzoek niet aan uw verwachtingen heeft voldaan
